# Isoniazid Induced Pure Red Blood Cell Aplasia

**DOI:** 10.7759/cureus.7112

**Published:** 2020-02-27

**Authors:** Waqas Azhar, Fawwad Zaidi, Abdul Hannan

**Affiliations:** 1 Hospital Medicine, Springfield Clinic, Springfield, USA; 2 Internal Medicine, Memorial Medical Center, Springfield, USA; 3 Internal Medicine, Saint John Hospital, Springfield, USA; 4 Internal Medicine, Southern Illinois University School of Medicine, Springfield, USA; 5 Hematology/Oncology, Southern Illinois University School of Medicine, Springfield, USA

**Keywords:** isoniazid, anemia, tuberculosis, pure red cell aplasia

## Abstract

Pure red blood cell aplasia (PRCA) is one of the uncommon causes of anemia. Drug-induced PRCA is even more infrequent. Only a few drugs are implicated in PRCA. Isoniazid is a widely used drug for the treatment of tuberculosis all over the world. It is known to cause hepatotoxicity, but in rare instances, it can lead to PRCA.

A 72-year-old Caucasian male, who was started on isoniazid after the diagnosis of latent tuberculosis, presented two months later with episodes of syncope to primary care physician’s office. The initial blood work showed severe anemia. There were no signs of acute or chronic gastrointestinal blood loss, and the stool hemoccult test was negative. Iron, vitamin B12, folate, lactate dehydrogenase, bilirubin, transaminases, and erythropoietin were within normal limits. Peripheral blood smear showed normochromic and normocytic anemia. A reticulocyte count was less than 1,000 per microliter. Thymoma, human immunodeficiency virus, and parvovirus B19 were ruled out. Further work-up with bone marrow biopsy confirmed pure red blood cell aplasia. A detailed review of recently started medications revealed isoniazid as the offending drug. Isoniazid was stopped. Reticulocyte count and bone marrow recovered a few days after stopping with eventual improvement in hemoglobin level, thus confirming the diagnosis of isoniazid induced red blood cell aplasia.

Isoniazid is the first-line therapy for tuberculosis. Rarely, it can cause pure red blood cell suppression and severe anemia, an untoward effect, worth remembering.

## Introduction

Pure red blood cell aplasia is an extremely unusual disorder, in which the patient presents with severe anemia, a marked reduction in circulating reticulocytes, and nonexistence of erythroid precursors in the bone marrow [1]. Causes of pure red blood cell aplasia vary from immunological disorders and infectious agents to hematologic malignancies. Infrequently drugs are implicated as the cause of pure red blood cell aplasia. Isoniazid is a first-line drug for the treatment of latent and active tuberculosis all over the world. The most common side effect of isoniazid is hepatotoxicity, but in very rare circumstances, it can lead to pure red blood cell precursor suppression.

## Case presentation

A 72-year-old male was admitted to the hospital three months before the current presentation for urinary tract infection, lumbar spinal stenosis, and failure to thrive. He was treated for infection and transferred to a rehabilitation facility. At the rehabilitation facility, he was evaluated for latent tuberculosis per their protocol with a Quantiferon gold test; the interferon-gamma release assay showed a value above the cutoff and was, therefore, interpreted as positive for tuberculosis exposure. A chest radiograph did not show any signs of active tuberculosis; neither did the clinical picture. The patient had latent tuberculosis. Infectious disease service was consulted, and the patient was started on isoniazid therapy for nine months. The patient tolerated isoniazid very well and was discharged after finishing his rehabilitation. At discharge, the patient’s hemoglobin was 13.6 grams per deciliter. A week after discharge, the patient had preoperative cardiac testing for his lumbar spine surgery. A pharmacological stress test showed reversible ischemia, and eventually, the patient received a bare-metal stent in the right coronary artery. The patient’s lumbar spine surgery was postponed secondary to coronary artery stenting.

**Figure 1:**
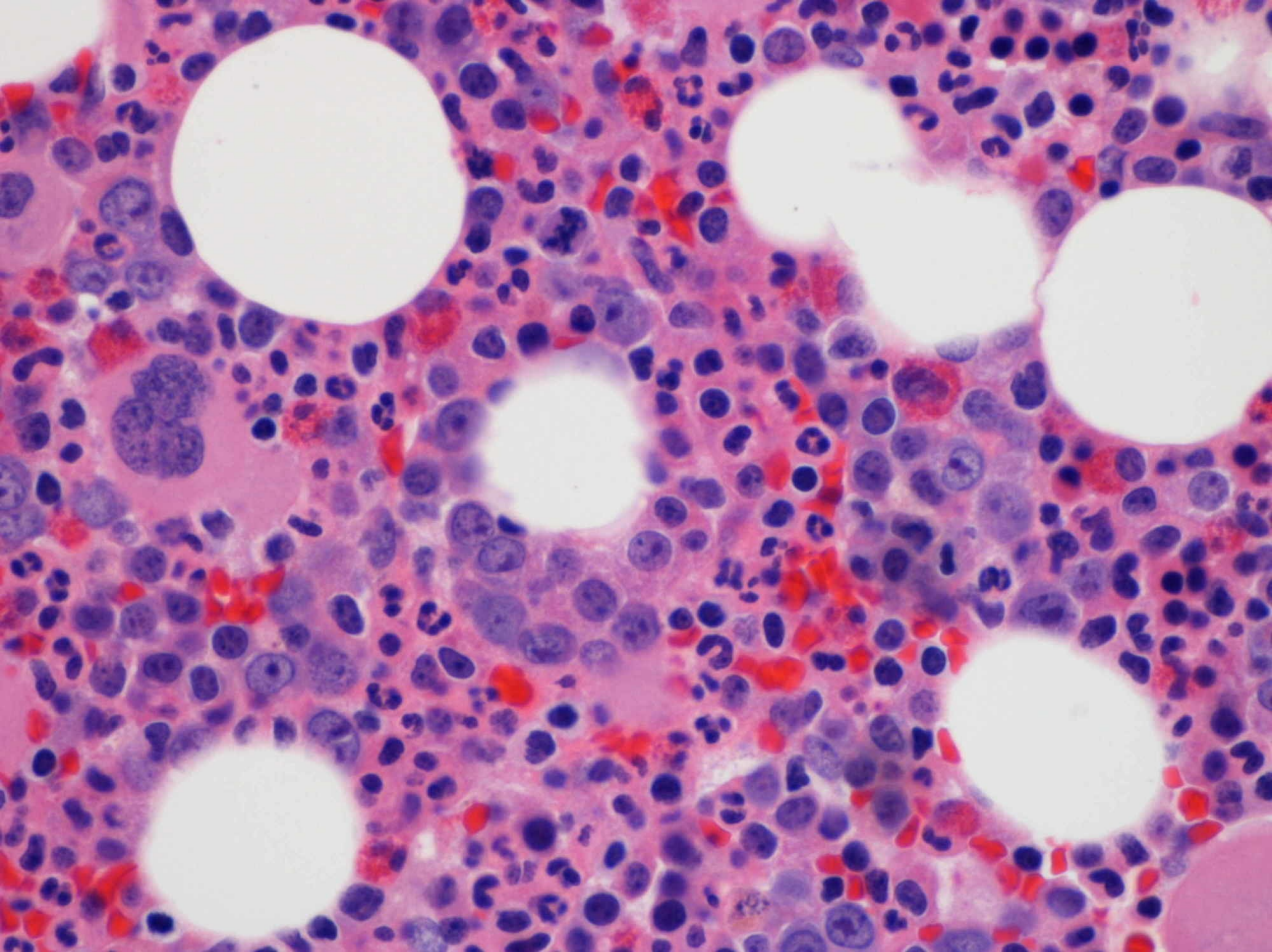
Bone marrow biopsy showing lack of erythroid precursors (Hematoxylin and eosin stain)

**Figure 2:**
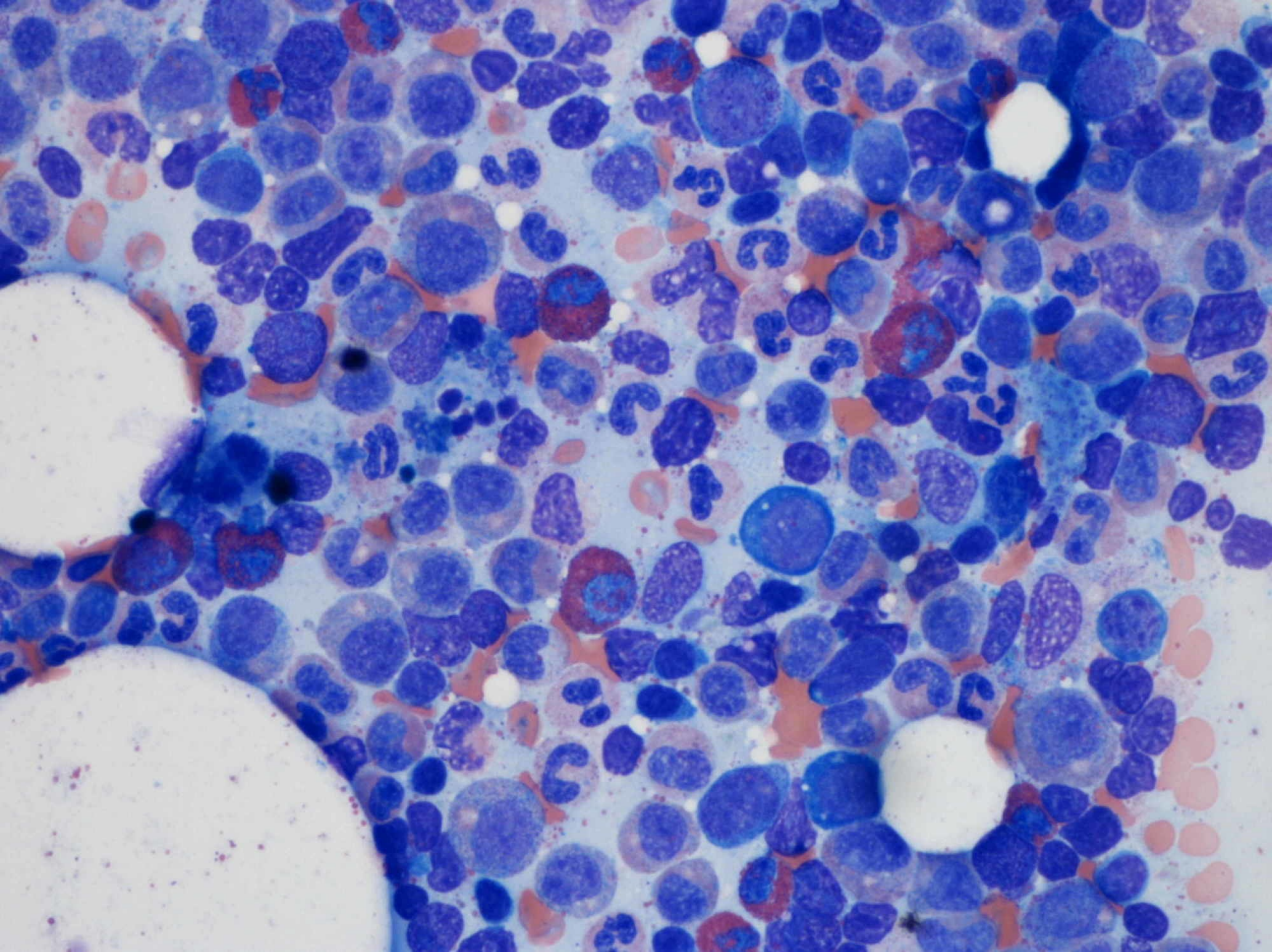
Bone marrow aspirate showing lack of erythroid precursors

Approximately two weeks before the presentation, the patient had episodes of dizziness and had an episode of syncope at home. No significant trauma occurred. The patient went to the primary care physician’s office for his symptoms of dizziness and falls, where basic lab work was performed, which showed severe anemia with hemoglobin of 5.8 grams per deciliter. Repeated laboratory blood work confirmed low hemoglobin. On detailed history and examination, there were no signs of blood loss. Stools were brown, and the stool hemoccult test was negative. Iron, vitamin B12, folate, lactate dehydrogenase, bilirubin, transaminase levels were within normal limits. Reticulocyte count was less than 1,000 per microliter. Peripheral blood smear showed normochromic and normocytic anemia and no other obvious pathology. The erythropoietin level was adequate. There was no history of synthetic erythropoietin. The patient had age-appropriate up-to-date malignancy screening, and there was no evidence of malignancy. Thymoma was ruled out by mediastinal imaging. Parvovirus B19, human immunodeficiency virus, and viral hepatitis were ruled out. Hematology service was consulted, and a detailed review of medications showed that the isoniazid is the suspected offending drug, causing anemia. White blood cell and platelet counts were in normal ranges. The patient was transfused with packed red blood cells with goal hemoglobin of 8.0 grams per deciliter, given coronary artery disease and recent coronary artery stenting. Isoniazid was stopped, and bone marrow biopsy and aspirate were obtained, which showed erythroid hypoplasia and arrest in the maturation phase (Figures 1, 2).

Megakaryocytic and myeloid precursors were normal. The patient’s symptoms of syncope and dizziness resolved after packed red blood cell transfusion. Further work-up for syncope, including computed tomography of the head, echocardiogram, carotid doppler, and cardiac telemetry monitoring, were unremarkable. The patient was discharged and had a periodic check of blood counts. The patient’s bone marrow showed signs of recovery on day 17 when reticulocyte count increased up to 50,000 per microliter, and hemoglobin reached 9.1 grams per deciliter. Later, the patient achieved hemoglobin of 10.3 grams per deciliter on day 31. Infectious diseases service recommended not to re-challenge the patient with isoniazid as the clinical picture was compatible with isoniazid induced pure red blood cell precursor suppression. The patient was started on rifampin for latent tuberculosis therapy after a few months.

## Discussion

Characteristic findings of PRCA include normocytic normochromic anemia with markedly depressed or near zero reticulocyte percentage and nonexistence of erythroid precursors in the bone marrow with the preservation of other cell lines. PRCA can be congenital in some cases, but mostly it is secondary to an acquired disease or condition. [1]. Some acquired conditions leading to PRCA include immune disorders (systemic lupus erythematosus, rheumatoid arthritis, ABO-incompatibility following bone marrow transplantation), malignancies (myeloma, chronic lymphoblastic leukemia, lymphomas, chronic myeloid leukemia, etc.), thymoma, chemotherapy-associated anemia, infections (parvovirus B19, human immunodeficiency virus), and infrequently drug-induced anemia [1,2]. Drugs account for less than 5% of cases of PRCA; few drugs mentioned in the literature known to cause PRCA are chloramphenicol, trimethoprim-sulfamethoxazole, phenytoin, and azathioprine [1-5]. The number of case reports demonstrating PRCA secondary to anti-tuberculosis treatment is scarce, but isoniazid has been cited as the root cause of PRCA in all the anti-tuberculosis treatment cases [1,3,6,7]. The major adverse effect of isoniazid is hepatotoxicity, and hematologic side effects are far less common. PRCA, hemolytic anemia, agranulocytosis, and sideroblastic anemia are among the significant hematologic abnormalities caused by isoniazid [8,9]. The pathogenesis and precise mechanism of isoniazid induced hematologic abnormalities, including PRCA, is not entirely clear. The immunologic phenomenon, for example, targeted cytotoxicity through lymphocytes, pointed disruption of DNA synthesis, antibodies mediated damage to red cell precursors or erythropoietin, and toxic suppression of the bone marrow are the postulated mechanisms mentioned in the literature [2,4,10,11]. After initiation of isoniazid, a time-lapse of up to six months may occur before any hematologic abnormalities can manifest, which explains and supports the immunologic phenomenon of PRCA [3]. Even though PRCA is a reversible condition, it can lead to critical illness as it easily remains undetected until the patient becomes symptomatic with severe anemia, as seen in our patient [1,3,6,11,12]. Discontinuation of the offending drug with periodic monitoring of blood counts is the mainstay of treatment. Immunosuppressive agents like corticosteroids, cyclosporine A or cyclophosphamide can be considered if anemia does not improve with discontinuation of the drug [5,13]. In most of the reported cases, anemia resolved rapidly within a short period, ranging from two to six weeks after the removal of the offending agent [5,6,8,11].

## Conclusions

Isoniazid is the first-line therapy for latent and active tuberculosis, especially amongst human immunodeficiency virus-infected individuals and the immigrant population. Hepatotoxicity is the most common adverse effect. In extremely rare instances, it can cause pure red blood cell suppression leading to severe anemia. It is, thus, imperative that physicians are at least aware of this rare but significant adverse effect. 
